# Mapping the rare disease paediatric clinical trial availabilities in Europe

**DOI:** 10.3389/fped.2025.1523847

**Published:** 2025-05-19

**Authors:** Eva Degraeuwe, Mark A. Turner, Ricardo M. Fernandes, Ann Raes, Johan Vande Walle, Franz Schaefer

**Affiliations:** ^1^ Department of Internal Medicine and Paediatrics, Faculty of Medicine and Health Sciences, Ghent University, Ghent, Belgium; ^2^ Department of Paediatric Nephrology, Ghent University Hospital, Ghent, Belgium; ^3^ Department of Pediatrics, University of Liverpool (ULIV), Liverpool, United Kingdom; ^4^ Laboratory of Clinical Pharmacology and Therapeutics, Faculty of Medicine, University of Lisbon, Lisbon, Portugal; ^5^ Zentrum für Kinder- und Jugendmedizin, Universitätsklinikum Heidelberg, Heidelberg, Germany; ^6^ Department of Pediatrics Nephrology, ERKNet: European Rare Kidney Disease Network, Heidelberg, Germany

**Keywords:** paediatric, drug development, networks, rare diseases c4c -ERN mapping clinical trial availabilities, network engagement

## Abstract

**Introduction:**

The prevalence and complexity of rare diseases (RDs) require concerted efforts in research and clinical trial capabilities. This paper aims to map the clinical trial sites within the Collaborative Network for European Clinical Trials for Children (conect4children, c4c) consortium and the European Reference Networks for Rare Diseases (ERNs), assessing their potential overlap and opportunities for synergies to optimize the selection and preparedness of sites for paediatric RD clinical trials.

**Method:**

A quantitative cross-mapping analysis was performed with publicly available data from ERN and c4c sites across 19 countries, complemented by information on paediatric site capabilities through interviews with network coordinators. Site analyses were done at country and setting levels. Heatmaps and an interactive matrix tool were developed using RStudio (v2023.12.0).

**Results:**

The highest overlap between ERN and c4c networks is found in the Netherlands, Belgium, Sweden, Denmark, and the Czech Republic, indicating strong integration in these regions, while Nordic (Sweden and Denmark), Eastern, and Southern European countries show varying levels of overlap. The median proportion of regional sites to University sites is 0.05 (IQR 0.12) across ERNs and 0.25 (IQR 0.37) across c4c national networks. The matrix tool can identify overlap and its absence for both university and regional hospitals, enhancing the preparedness and reach of paediatric rare disease trials. ERN representatives confirm the heatmap and matrix tool's utility in improving site selection and fostering network cooperation.

**Conclusion:**

Heatmap analyses reveal a significant but incomplete overlap of RD clinical trial sites between ERNs and c4c in parts of Europe, suggesting strong potential for cross-network collaboration to enhance paediatric RD trial recruitment and outcomes.

## 1 Introduction

Rare diseases (RDs) are a public health priority, including over 6,000–8,000 unique RDs globally (1–3). The European Union Regulation on orphan medicinal products defined RDs as affecting less than 50 per 100,000 individuals, or roughly 1 in 2,000 individuals (4). Even though each disease is classified as rare, the collective number of individuals with RDs is equivalent to the population of the world's third largest country (5, 6). Up to 75% of RDs have a paediatric onset (1, 2).

Currently, around 95% of these RDs are lacking an approved therapy (7). Increased engagement with rare diseases and focused initiatives over the last decades have aimed to spur development in this area by incentivizing pharmaceutical and biotechnology companies to develop appropriate therapies (8–10). Since the launch of the Orphan Drug application and Orphan Medicines (EC/131/2000) in 2000, 82 orphan drugs have been approved in the EU (8, 11). Aside from the EU, the WHO has created the Global Accelerator for Paediatric Formulations Network GAP-f for prioritising new and existing Paediatric Formulations (12). Orphan drugs are predicted to account for 20% of global prescription revenue by 2024, with an expected annual growth rate of 9.7% from 2023 to 2030 (13, 14). However, investments in orphan drug development are threatened by the high failure rate of clinical trials, particularly in paediatric settings, where up to 42% of paediatric drug development programmes fail to establish a new or expanded paediatric indication (15).

The burden of living with a rare disease is not limited to limited therapeutic options. Many patients are only diagnosed after a long diagnostic odyssey (16–18) associated with ineffective or unnecessary medical management (18). Recognizing the need to improve healthcare for patients with rare diseases, the EU established 24 thematic European Reference Networks for rare diseases (ERNs) in 2017, as shown in Table 1 (19, 20). The objective of the ERNs is to harmonize and optimize RD healthcare throughout the EU by facilitating patient access to the best expertise, developing clinical guidelines and decision-supporting tools, educating healthcare professionals, forming EU-wide patient registries and fostering clinical research projects (17, 19). The ERNs connect more than 1,600 highly specialized units at 332 healthcare providers across Europe, selected in a rigorous application and evaluation procedure.

**Table 1:** Overview of ERNs created to improve the clinical care for rare diseases.

ERN name	Thematic area	Website showcasing sites involved
Endo-ERN	European Reference Network on endocrine conditions	https://endo-ern.eu/reference-centres/
EpiCare	European Reference Network on epilepsies	https://epi-care.eu/members-of-epicare/
ERKNet	European Reference Network on kidney diseases	https://www.erknet.org/our-experts/the-european-reference-centers
ERN BOND	European Reference Network on bone disorders	https://ernbond.eu/
ERN CRANIO	European Reference Network on craniofacial anomalies and ear, nose and throat (ENT) disorders	https://www.ern-cranio.eu/
ERN Euracan	European Reference Network on adult cancers (solid tumours)	https://euracan.eu/who-we-are/members-and-partners/
ERN Euro-NMD	European Reference Network on neuromuscular diseases	https://ern-euro-nmd.eu/hcps/
ERN EuroBloodNet	European Reference Network on haematological diseases	https://eurobloodnet.eu/members/
ERN eUROGEN	European Reference Network on urogenital diseases and conditions	https://eurogen-ern.eu/who-is-involved/healthcare-providers/
ERN EYE	European Reference Network on eye diseases	https://www.ern-eye.eu/ern-eye-members
ERN GENTURIS	European Reference Network on genetic tumour risk syndromes	https://www.genturis.eu/l=eng/Our-experts/Our-healthcare-providers.html
ERN GUARDHEART	European Reference Network on diseases of the heart	https://guardheart.ern-net.eu/
ERN ITHACA	European Reference Network on congenital malformations and rare intellectual disability	https://ern-ithaca.eu/about-us/expert-centers/
ERN LUNG	European Reference Network on respiratory diseases	https://ern-lung.eu/about-ern-lung/reference-centers/
ERN PAEDCAN	European Reference Network on paediatric cancer (haemato-oncology)	https://paedcan.ern-net.eu/home/member-institutions/
ERN Rare Liver	European Reference Network on hepatological diseases	https://rare-liver.eu/about/members-and-partners-of-the-network
ERN ReCONNET	European Reference Network on connective tissue and musculoskeletal diseases	https://reconnet.ern-net.eu/our-network-members/
ERN RITA	European Reference Network on immunodeficiency, autoinflammatory and autoimmune diseases	https://ern-rita.org/hcps/
ERN SKIN	European Reference Network on skin disorders	https://ern-skin.eu/reference-centers/
ERN TRANSPLANTCHILD	European Reference Network on Transplantation in Children	https://transplantchild.eu/about-transplantchild/
ERN-RND	European Reference Network on neurological diseases	https://www.ern-rnd.eu/expertcentres/
ERNICA	European Reference Network on inherited and congenital anomalies	https://www.ern-ernica.eu/
MetabERN	European Reference Network on hereditary metabolic disorders	https://metab.ern-net.eu/about-us-3/#centers
VASCERN	European Reference Network on Rare Multisystemic Vascular Diseases	https://vascern.eu/network-people/experts-centers/centers/

The facilitation of multinational paediatric trials has been a growing need since the 2007 Paediatric Regulation and the subsequent increase in paediatric clinical trial activity (21–23). To optimize and facilitate paediatric trials in Europe, the Innovative Medicines Initiative has funded a large project that established the conect4children (c4c) consortium aimed at creating a pan-European clinical trial network. Twenty countries have joined the consortium by establishing National Hubs, the majority hosted by already established National paediatric clinical trial Networks (24) supporting and developing paediatric trial units within relevant hospitals (22, 23). In addition to providing a uniform infrastructure facilitating the coordinated execution of clinical trials in experienced paediatric centres, the c4c Network provides sponsors with unique methodological and clinical expertise to support paediatric drug development and trial design in all paediatric subspecialties (22, 25–27). A non-profit organization has recently been incorporated to ensure the sustainability of the work of the consortium.

The c4c Network and the ERNs were constructed with different objectives and organizational concepts. However, both are based on mostly academic European hospitals and share a common focus on clinical research. Considering the high need for therapeutic trials in the field of RDs and the large proportion of children and adolescents in the RD population, the mapping of c4c-supported trial-ready specialized sites both within and outside the ERN expert centres is of potential interest both to academic investigators and industry sponsors. This mapping may help to expedite the selection of qualified sites for clinical trials and avoid redundant efforts. In this work, we set out to (1) evaluate and map the level site overlap between both networks through heatmaps, (2) develop an interactive matrix tool where ERN coordinators and investigators can map the state of paediatric clinical trial preparedness within and beyond their networks in detail, and (3) explore the utility, and needs for improvement, of the heatmaps and interactive matrix tool.

## 2 Methodology

To map the landscape of paediatric trial sites for RD research in Europe, a quantitative cross-mapping analysis was performed and a tool for interactive mapping per ERN was developed.

### 2.1 Data collection and cleaning

The mapping occurred in a stepwise manner, first at the national level, then at individual site levels. Eligible countries were those engaged with both c4c and ERNs. Exclusion criteria for ERNs included the absence of paediatric units (ERN Genturis, ERN EURACAN), and the UK (being only involved in c4c and not in the ERNs). A modification that has been performed *a posteriori* is the exclusion of the ERN PAEDCAN, considering the advancement of the oncology network of ITCC (innovative therapies for children and adolescents with cancer) and SIOP Europe (the European Society for Paediatric oncology) and inapplicability of the mapping to the field of oncology (28, 29). ERN membership requires clinical centers to demonstrate their specific expertise and capacity in a rigorous application process followed by annual monitoring according to the EU directive (3.2014/287/EU). A site is connected within c4c by expert analysis of paediatricians of each country through an application form investigating trial preparedness, experience and facilities on site (30, 31).

The initial site data was collected in November to December 2023 using only information from the public domain on the c4c paediatric network or ERN websites. Countries were identified using the ISO ALPHA-2 code country tag. The data was collected using Excel (v2.73, Microsoft Corporation 2023) within a Master Site List (MSL). The MSL contained separate tabs for ERNs and c4c data collection. Parameters per tab collected included: (i) network associated (c4c and/or ERN connected); (ii) country; (iii) site name; (iv) paediatric units; (iv) university or regional hospital.

The public sources of data had a number of shortcomings that required protocol adjustments and data verification using additional sources. When reviewing various European Reference Networks (ERNs) online, there were multiple discrepancies in site naming. For example, one Italian site had seven different names. Site names were standardized for our MSL by using one name per HCP to avoid duplicates. Data on ERN sites often didn't clarify if a paediatric Health Care Provider (pHCP) representative was available, ie either a representative officially connected with the ERN with a paediatric background or performing regular (weekly) consultations with paediatric patients. The existence of Paediatric HCPs were confirmed by website and emailing ERN coordinators and managers from beginning-of-December 2024 until end-of-August 2025. One ERN was excluded due to persistent unresponsiveness. Data on c4c sites needed to complement publicly available information was obtained through direct contact with the c4c national networks during December 2024 until April 2025.

Overlap was defined as having an identical site name per country between an ERN specifically and the respective paediatric national network.

For our interactive matrix tool, we incorporated data from both university and regional sites. University sites were defined as sites with a connected university and/or which were recognized either in title or through hospital site verification. University sites provide tertiary care, required in most RD cases. Regional sites were defined as sites that provide secondary care in an almost exclusively urban setting and which were not recognized as university sites either by name or through their website and/or did not have a university connected to the hospital directly. In analysing these sites within the c4c national networks and their representation in ERNs, RStudio (v2023.12.0) was used to calculate the following proportions: i) the number of regional hospitals of all ERNs per country (corrected for duplicates) divided by the total number of sites; and ii) the number of regional hospitals per c4c country network divided by the total number of sites.

### 2.2 Heatmap analysis of overlapping sites

A total of four heatmaps were produced. Our aim was to assess the degree of site overlap across the networks, out of the total number of sites in each network. Therefore, we created heatmaps showing both absolute numbers (total amount of ERN paediatric sites and total amount of overlapping sites per ERN and per country), as well as relative numbers (when compared to all paediatric ERN sites and when compared to all c4c university sites).

The first heatmap set showcased the absolute numbers of sites that are included in both ERNs and c4c, ie overlapping sites, per country and per ERN.

The second heatmap displayed data from Equation 1, calculating the number of overlapping sites divided by the number of paediatric sites per ERN in each country, shown as a percentage (%).

(1) $$(1)=\;\frac{Number\;of\;overlapping\;c4c\;sites\;and\;ERN\;sites\;per\;country}{Number\;of\;paediatric\;sites\;per\;country\;per\;ERN}\;\;\times \;\;100$$

The third heatmap set visualized data from Equation 2, calculating the number of overlapping sites divided by the number of university sites of each c4c national network per each country, shown as a percentage (%). We chose to focus on university sites for a more homogenous comparison, since the number of regional sites were found to be lower than the number of c4c regional sites.

(2) $$(2)=\;\frac{Total\;number\;of\;c4c\;sites\;and\;ERN\;sites\;per\;country\;}{Total\;number\;of\;c4c\;university\;sites\;per\;country}\;\;\times \;\;100$$

To describe the extent of inclusion of regional (non-university) sites (for both the c4c and ERN networks), a per country calculation was performed shown by Equation 3. From all results across each network, we described a median and IQR.

(3) $$(3)=\frac{\begin{array}{rl} & number\;of\;regional\;sites\;per\;country\;(included\;in\;c4c\;OR\\ & \;\;\;included\;in\;at\;least\;one\;ERN) \end{array}}{\begin{array}{rl} & total\;sites\;per\;network\;(included\;in\;c4c\;OR\\ & \;\;\;included\;in\;at\;least\;one\;ERN) \end{array}}$$

The heatmaps are ordered by the average of the row and column of the heatmap in ascending order. The average per ERN calculations for all countries are shown through descriptive analysis by average counts and percentages (%). The description of the heatmap's order in countries is in ascending order (highest to lowest) for the top or lowest five ranked countries or ERNs. Per heatmap a median and IQR is calculated of the overlap across each network.

The dataset was analysed using RStudio (v2023.12.0) using packages “*readxl*”, “*dplyr*”, “*tidyr*” “*ggplot2*” and “*reshape2*”.

### 2.3 Interactive matrix tool development

An interactive matrix tool was developed for ERN coordinators and project managers to evaluate the status of their network per country and compare it to the c4c network.

The tool was developed using RStudio (v2023.12.0) using packages “*shiny*”, “*DT*”, “*dplyr*”, “*readxl*” and “*shinyjs*”, Shiny R web application (v1.8.0) and written in JavaScript. The data source was the file containing all the ERNs site and c4c sites combined in two sheets for the application programming interface construction. The user interface incorporated selection of ERNs and countries, displaying of an adaptive matrix of network details and site overlap.

### 2.4 Heatmap and tool value proposition

To gain insights into the interpretation and value of the heatmaps and Interactive matrix tool, one author (EVD) conducted qualitative semi-structured remote interviews with coordinators and/or project managers from the 22 included ERNs between 2 and 3/2024. These interviews utilized a prompt guide and assessed the relevance of the study's heatmap and tools for each ERN separately.

Interviews were conducted over Microsoft Teams (v2023.44.01.07), enabling session recordings and transcriptions, which were then coded and thematically analysed to identify key themes. Session recordings and subsequent transcriptions were subjected to a systematic coding process, wherein segments of the text were labelled with specific codes. These codes were generated both deductively, based on predefined theoretical frameworks, and inductively, allowing themes to emerge directly from the data. The coding process facilitated the organization and interpretation of the dataset, enabling the identification of key patterns and recurring ideas. An independent review by a second researcher (MAT) was implemented to validate the data interpretation. The research involved administrative, non-personal data, waiving the need for an ethics committee review.

## 3 Results

**Figure 1:**
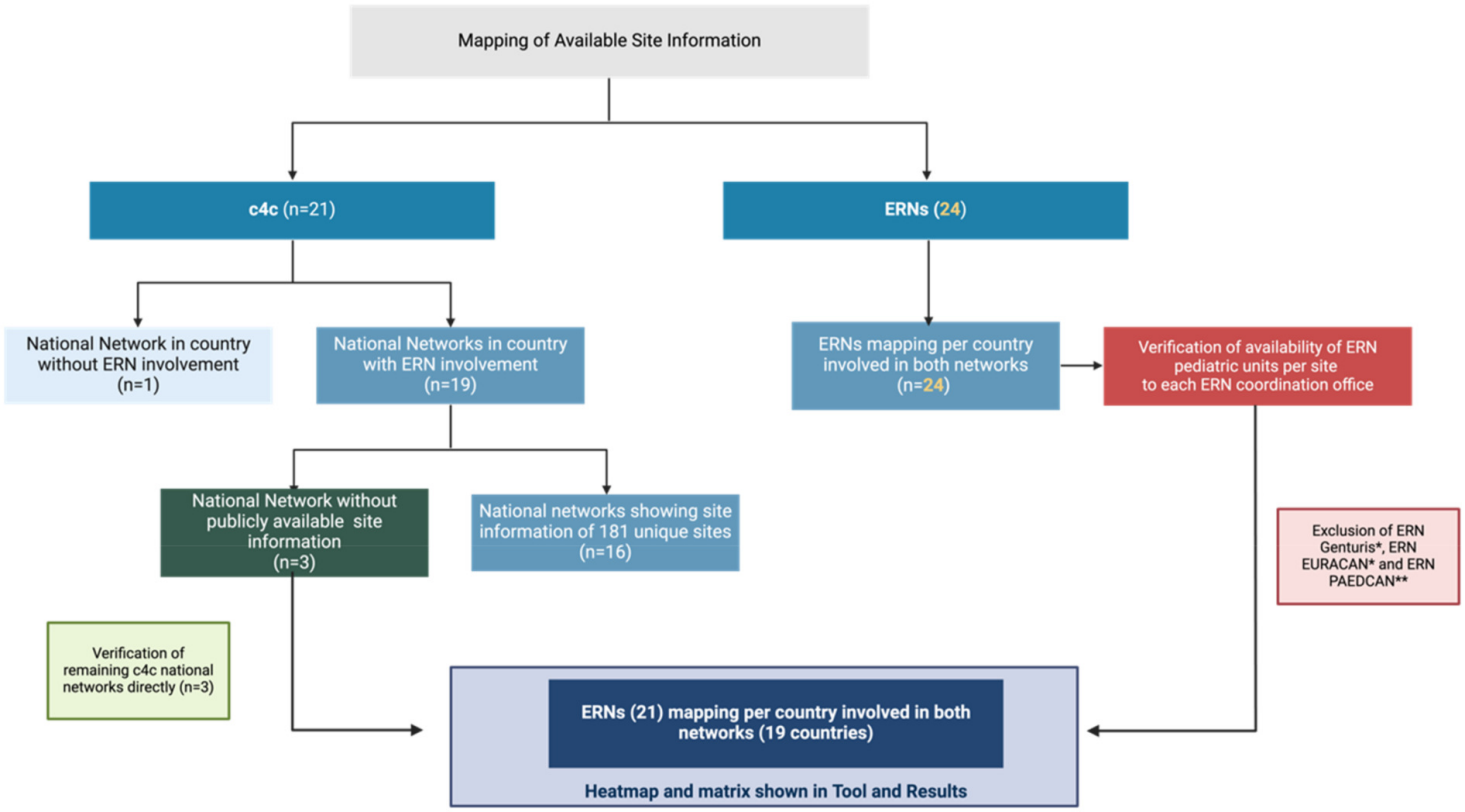
Mapping of available site information. The blue boxes include the publicly available information. The red box indicates where non-public available information had to be requested from the coordinators. The green boxes identifies cases where verification of non-public member sites for the three c4c national networks was required. The dark blue box with cross mapping analysis shows the source data basis for the site identification mapping between c4c and ERNs, including paediatric-oriented HCP, removal of non-paediatric or not applicable ERNs, for both adult and paediatric as well as paediatric only. Within the c4c networks, one network is representing two countries. *ERNs that do not see paediatric patients. **ERN not applicable for use within the c4c structure due to oncology focus. c4c, conect4children consortium; ERN, European Reference Network. Created with BioRender.com (2023).

The flowchart of mapping and quantitative paediatric cross mapping is shown in Figure 1.

**Figure 2:**
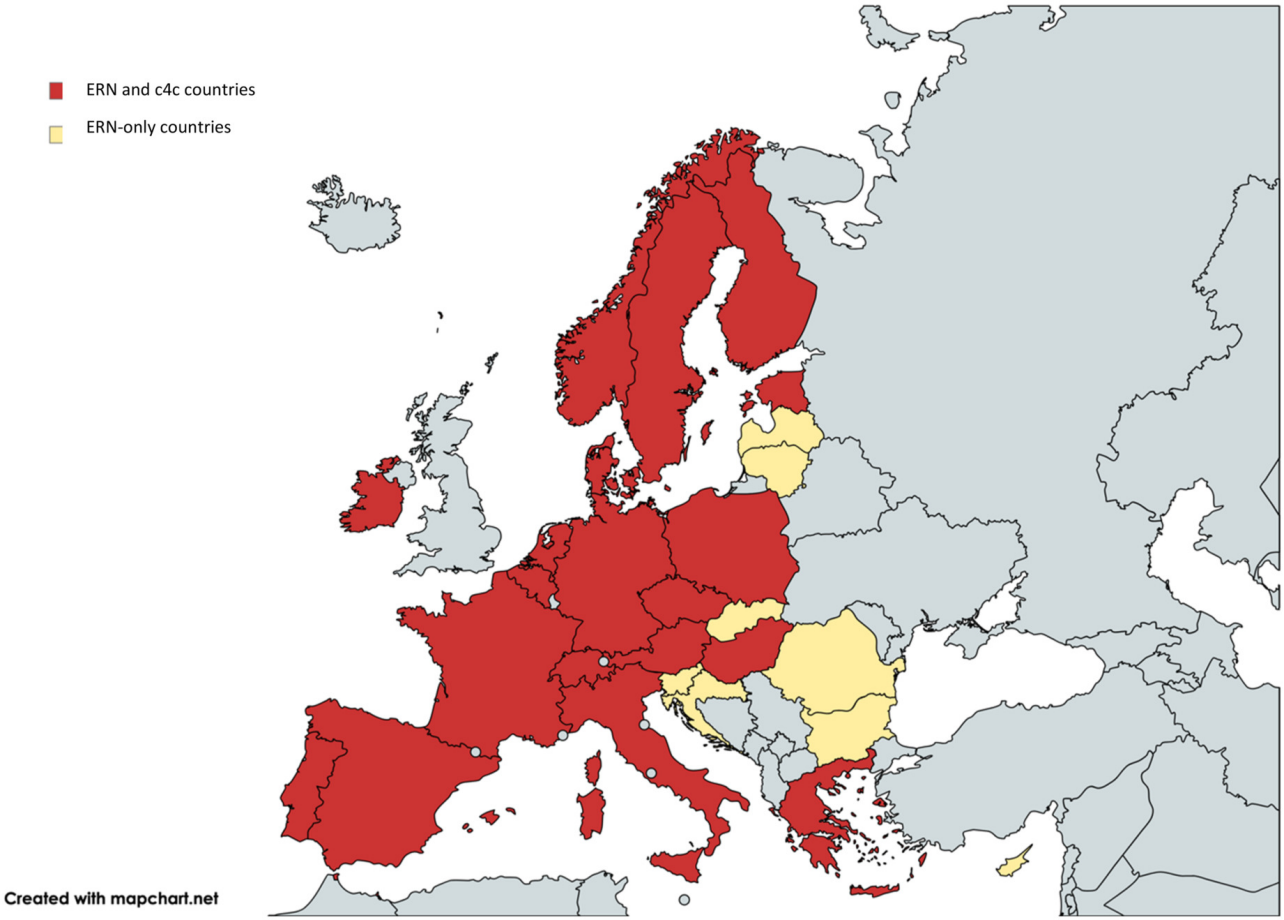
Overview of overlapping countries between c4c and ERN. Countries included in both ERNs and c4c are shown in red. The countries that do not have a c4c national network are shown in yellow. The UK is a national network within c4c, however, does not showcase its involved sites and is shown in blue. One ERN involves the UK yet is not a standardly included country in the ERNs. c4c, conect4children consortium; ERN, European Reference Network. Created with Mapchart.net (2024).

Figure 2 shows the countries involved in both networks, including a total of 317 sites in 19 countries. The c4c national networks and their relevant websites or site information are shown in Table 2. The countries that are ERN-only are mostly located in Eastern Europe. Therefore, a total of 19 countries including regional and university paediatric sites were mapped through heatmaps and incorporated within the interactive matrix tool.

**Table 2:** Overview of countries involved in both ERN and c4c, including national network for c4c paediatric clinical trials, with connected website and site network availability.

Included in both ERN and c4c	National network	Site of the national network	Showing connected Sites Publicly (Yes = 1, No = 0)
Austria	Okids	https://www.okids-net.at/de	1
Belgium	BPCRN	https://www.bpcrn.be/bpcrn-clinical-trial-sites	1
Czech Republic	CzechPharmNet	https://cuni.cz/UKEN-950.html	0
Denmark	DanPedMed	https://trialnation.dk/wp-content/uploads/2023/08/Detailed-overview-of-research-strategies-and-research-units-aug_2023.pdf; https://trialnation.dk/professional/resources/	1
Estonia	ELAV	https://www.elav.ee/partners	1
Finland	FINPedMed	https://finpedmed.fi/en/clinical-trial-units/university-hospitals/	1
France	PedStart	https://www.pedstart.org/le-reseau/les-centres-du-reseau	1
Germany	GermanNetPaet	https://www.germannetpaet.de/about/study-centres	1
Greece	HelpNET	https://helpnet.gr/members/	1
Hungary	MRCN	https://mcrn.hu/en/	0
Ireland	In4Kids	https://in4kids.ie/	1
Italy	InciPiT	https://www.incipit-pediatric.net/en/	1
Netherlands	Pedmed-NL	https://pedmed.nl/about-us/consortium-members/	1
Norway	NorPedMed	https://www.norcrin.no/en/contact-norcrin/norcrin-partners/	1
Poland	PolPedNet	https://nauka.czd.pl/badania-kliniczne/polska-siec-badan-klinicznych-w-pediatrii-polpednet/	1
Portugal	Stand4Kids	https://www.stand4kids.pt/organization/research-centers-map/	1
Spain	Reclip	https://reclip.org/organization.html	1
Sweden	SwedPedMed	https://www.swedpedmed.se/	0
Switzerland	SwissPedNet	https://www.swisspednet.ch/home	1

ERN, European Reference Network; c4c, conect4children; N/A, not available.

### 3.1 Heatmap of overlapping sites

**Figure 3:**
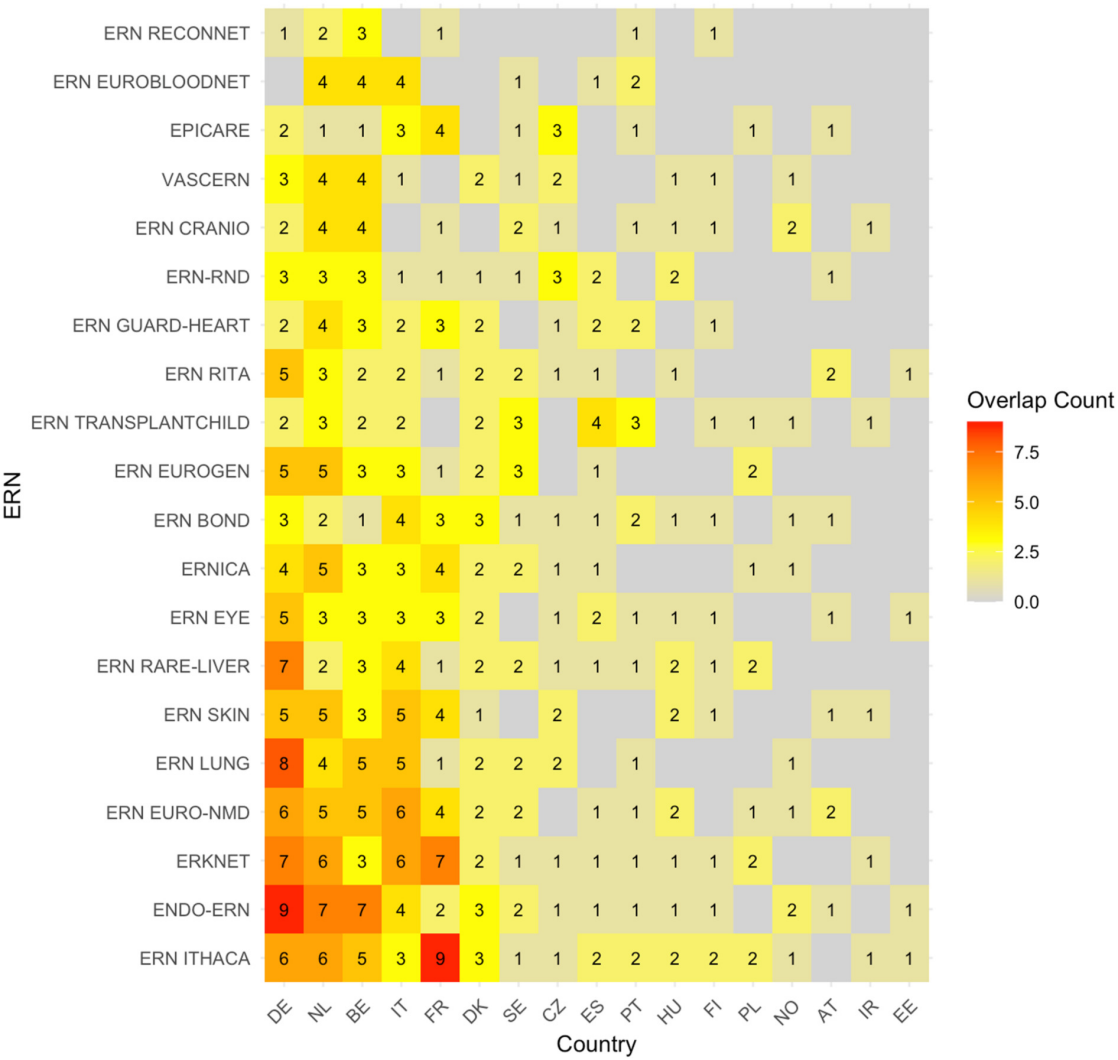
Heatmap according to absolute numbers showing overlap between c4c-ERN paediatric health care provider (HCP) per country per ERN. Colour spectrum is ordered by grey (no inclusion), to yellow and orange, and the highest in light green. Each row and column are ordered by highest average and on an ascending order. c4c, conect4children consortium; ERN, European Reference Network. For ERN acronyms please see Table 1. For the country codes, please the ISO ALPHA-2 codes.

The heatmap showing the absolute number of overlapping sites between c4c and ERN infrastructure is shown in Figure 3. The countries with the highest overlap in sites were Germany, Netherlands, Belgium, Italy, and France. The countries with the lowest overlap in sites were Poland, Norway, Austria, Ireland, and Estonia. Moreover, the countries with a middle-ranking in overlapping sites were Spain, Portugal, and Czech Republic. The ERNs with the highest overlap in sites were ERN ITHACA, Endo-ERN, ERKNet, ERN EURO-NMD and ERN LUNG. The ERNs with the lowest overlap in sites were in ERN ReCONNET, ERN EuroBloodNet, EpiCare, VASCERN and ERN CARDIO.

**Figure 4:**
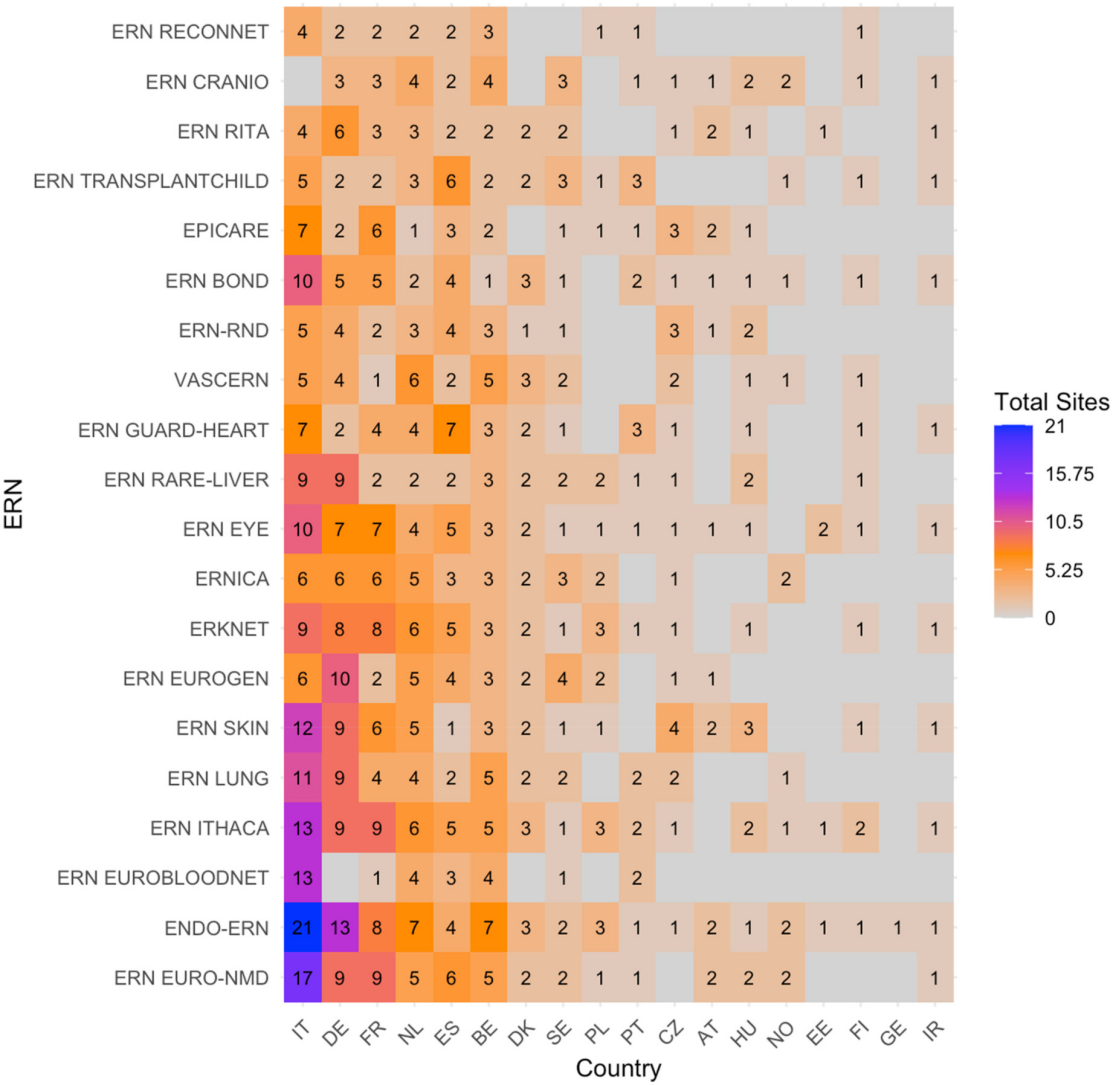
Heatmap according to absolute numbers showing the total amount of paediatric health care provider (HCP) sites within the ERNs. Colour spectrum is ordered by grey (no inclusion), to orange and red, and the highest in purple. Each row and column are ordered by highest average and on an ascending order.c4c, conect4children consortium; ERN, European Reference Network. For ERN acronyms please see Table 1. For the country codes, please the ISO ALPHA-2 codes.

The heatmap with absolute number of sites per country per ERN (without considering c4c sites) is shown in Figure 4. The countries with the highest number of sites per ERN were similar to those with the highest overlap with the paediatric networks, except for Denmark, Sweden and Czech Republic. The ERNs with the highest amount of paediatric sites were also similar, and include ERN EuroBloodNet (3rd) additionally.

**Figure 5:**
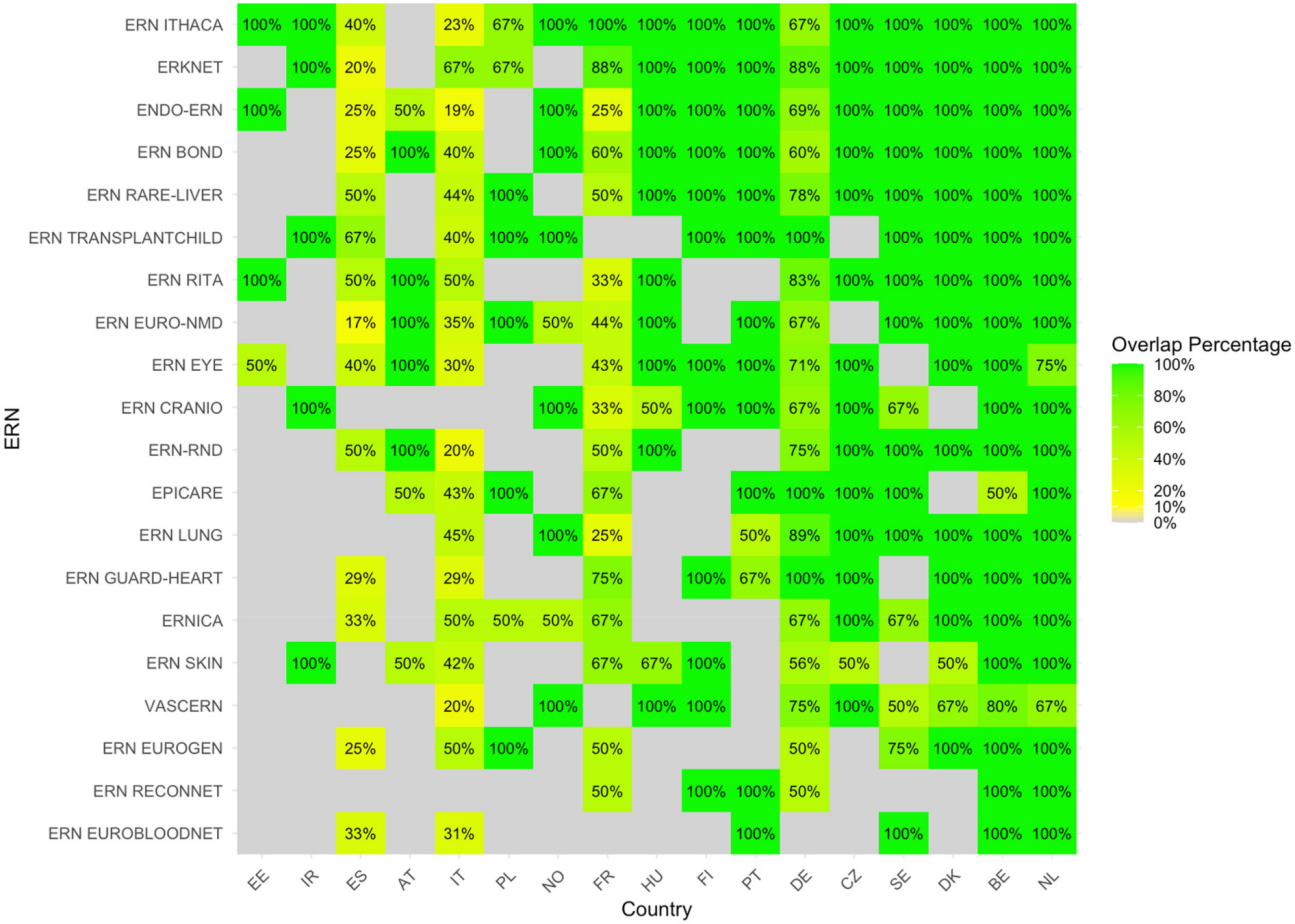
Heatmap according to relative numbers showing overlap between c4c-ERN paediatric health care provider (HCP) per country per ERN on the total amount of HCP sites within the ERNs, according to formula (1). Colour spectrum is ordered by grey (no inclusion), red (having no overlapping sites), to orange and red, and the highest in green. The median overlap is 100% with IQR of 33%. Ordered by highest average for both column and row on an ascending order. c4c, conect4children consortium; ERN, European Reference Network. For ERN acronyms please see Table 1. For the country codes, please the ISO ALPHA-2 codes.

The heatmap with the proportion of overlapping sites between c4c and ERN infrastructure calculated by Equation 1 is shown in Figure 5. The countries with the highest relative overlap when compared to the total amount of ERN sites were Netherlands, Belgium, Denmark, Sweden and Czech Republic. The countries with the lowest proportional overlap between c4c and ERN when compared to all ERN sites included Italy, Austria, Spain, Ireland and Estonia. The ERNs with the highest relative overlap when compared to the total amount of ERN sites are ERN ITHACA, ERKNet, Endo-ERN, ERN BOND and ERN Rare-liver. The ERNs with the lowest relative overlap are ERN SKIN, VASCERN, ERN EUROGEN, ERN ReCONNET and ERN EuroBloodNet.

**Figure 6:**
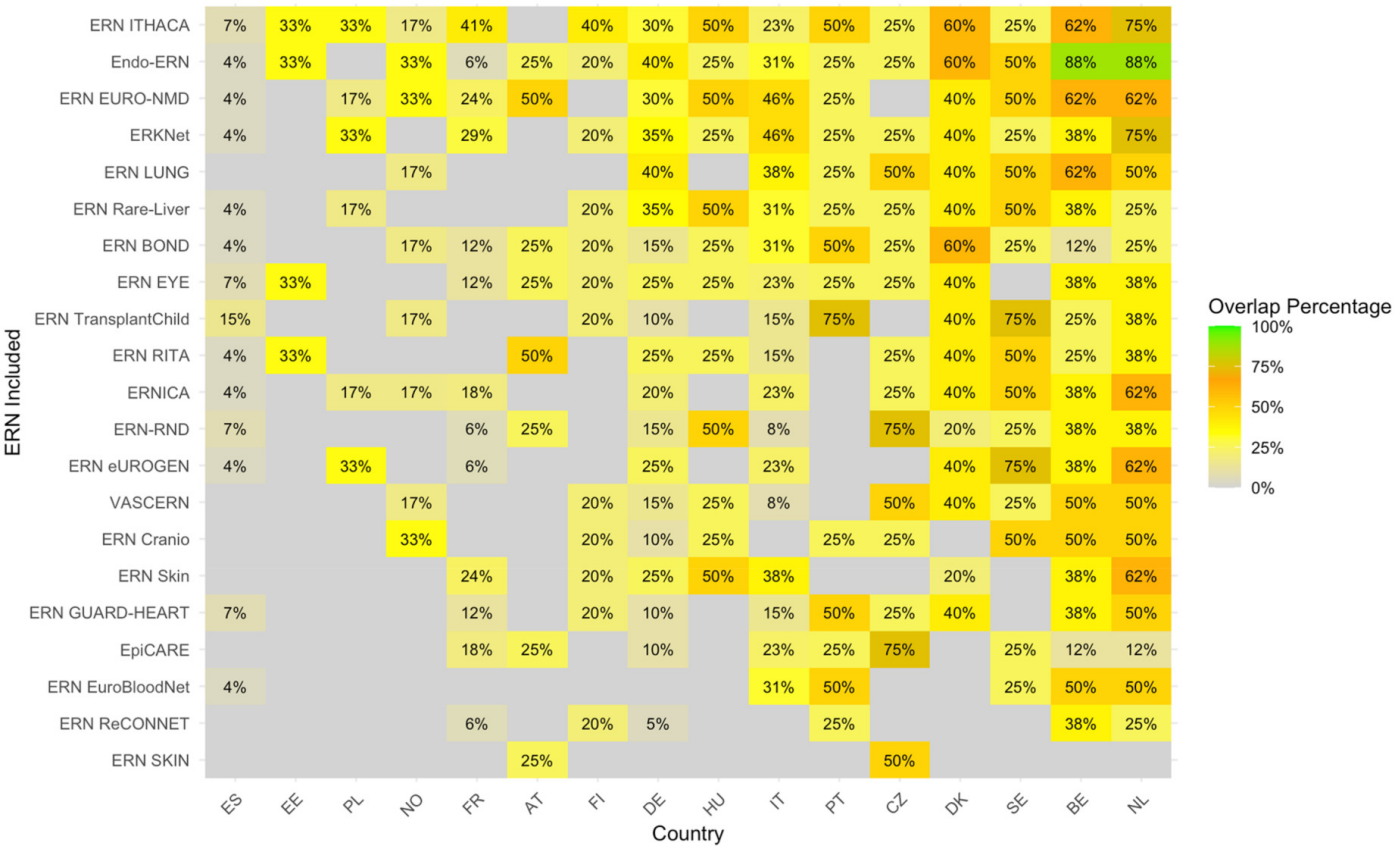
Heatmap according to relative numbers showing overlap between c4c-ERN paediatric health care provider (HCP) per country per ERN on the total amount of c4c university sites per country [according to formula (2)]. Colour spectrum is ordered by grey (no inclusion), to orange and red, and the highest in green. Ordered by highest average for both column and row on an descending order. Median overlap is 33% with IQR 20%. c4c, conect4children consortium; ERN, European Reference Network. For ERN acronyms please see Table 1. For the country codes, please the ISO ALPHA-2 codes.

The heatmap with the proportion of overlapping sites between c4c and ERN infrastructure calculated by Equation 2, i.e., based on university sites, is shown in Figure 6. The countries with the highest relative overlap when compared to all c4c university sites are Netherlands, Belgium, Sweden, Denmark and Czech Republic. The countries with the lowest overlap when compared to all c4c university sites are France, Norway, Portugal, Estonia and Spain. The ERNs with the highest relative overlap when compared to all c4c university sites were ERN ITHACA, Endo-ERN, ERN EURO-NMD, ERKNet, and ERN LUNG. The ERNs with the lowest relative overlap were ERN Guard-Heart, ERN EpiCARE, ERN EuroBloodNet, ERN ReCONNET and ERN SKIN.

**Figure 7:**
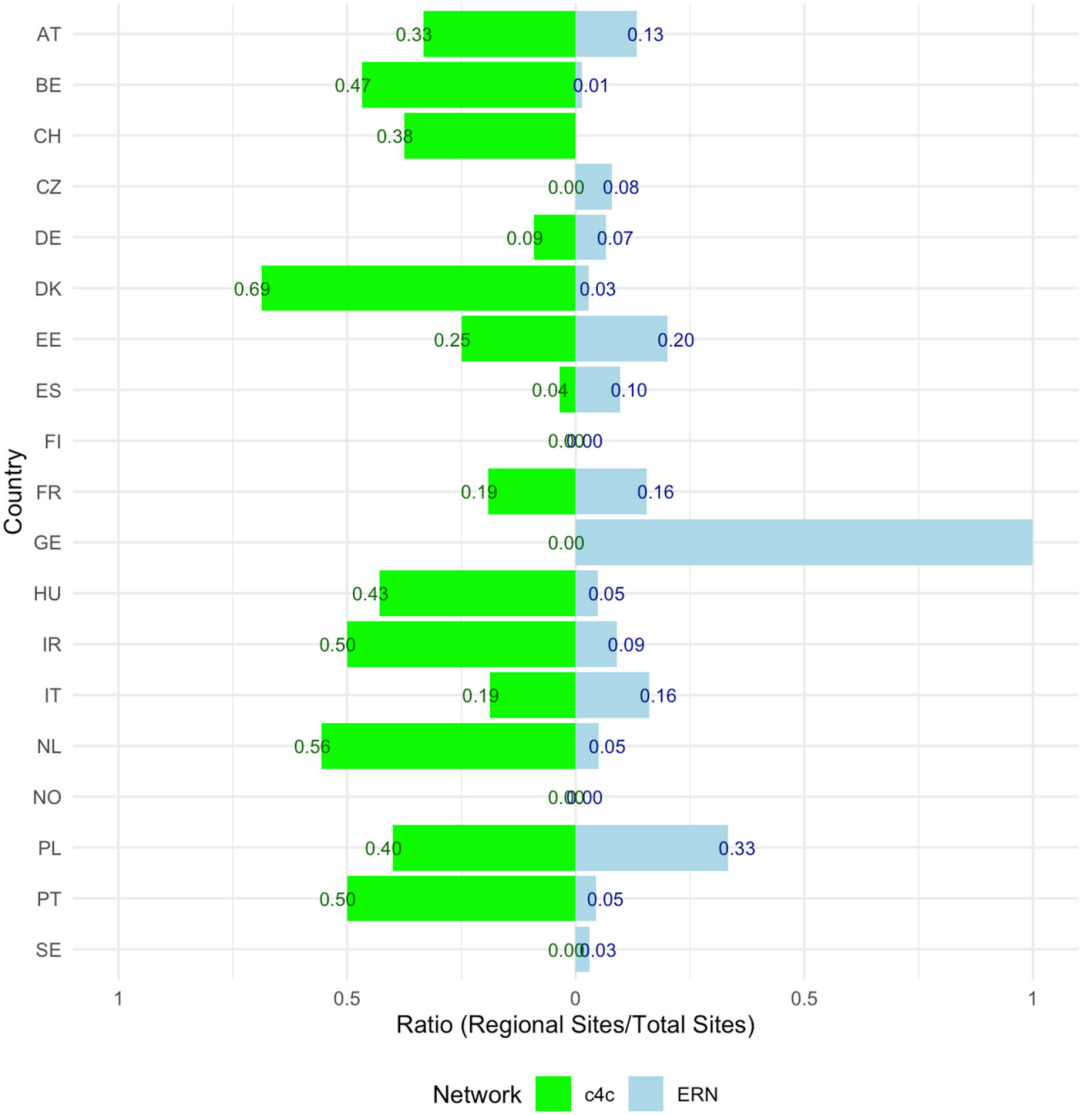
Side Bar chart showing the proportion (0–1) of paediatric regional sites connected in the ERNs vs. c4c, including all ERNs per country and corrected for duplicated site names. The ratio of regional inclusion for ERNs has a median of 0.05 (IQR 0.12). The ratio of regional inclusion for c4c networks has a median of 0.25 (IQR 0.37) as calculated by formula 3. The countries Finland and Norway have no regional sites included within both c4c and ERNs. For Greece, all the sites connected over the ERNs are regional hospitals. In the Czech Republic, no regional hospitals are connected in the c4c network. c4c, conect4children consortium; ERN, European Reference Network. For the country codes, please the ISO ALPHA-2 codes.

The side bar chart in Figure 7 shows the proportion or regional sites connected in the ERNs vs. c4c per country. Within the c4c networks there is a higher regional site inclusion for 79% of the countries (*n* = 15). The ratio of regional inclusion for ERNs has a median of 0.05 (IQR 0.12). The ratio of regional inclusion for c4c networks has a median of 0.25 (IQR 0.37) as calculated by Equation 3. The countries Finland and Norway have no regional sites included within both c4c and ERNs. For Greece, all the sites connected over the ERNs are regional hospitals. In the Czech Republic, no regional hospitals are connected in the c4c network.

### 3.2 Interactive matrix tool

**Figure 8:**
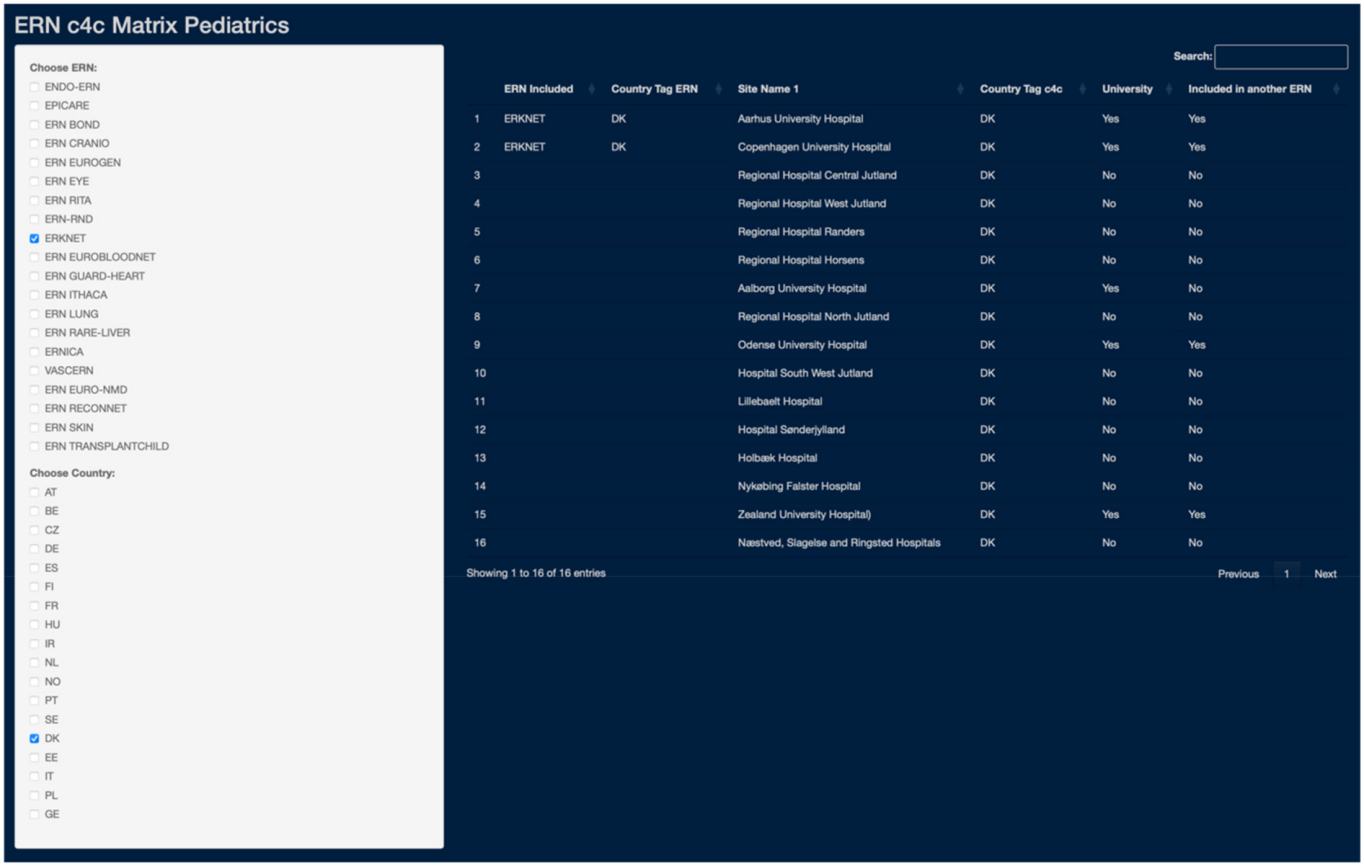
Use-case of the ERN and c4c interactive matrix: an example of ERKNet in Denmark (DK). The matrix use-case showcases the integration of ERKNet paediatric sites within Denmark (DK), detailing connections by country, site names, c4c inclusion with respective country tags, site type distinction (University as “Yes”, Regional as “No”), and cross-ERN participation. The matrix tool can be used to analyse collaborative networks and paediatric clinical trial capacities through c4c connection. c4c, conect4children consortium; ERN, European Reference Network. For ERN acronyms please see Table 1. For the country codes, please the ISO ALPHA-2 codes.

The interactive matrix, developed for internal use, was designed for the use of an ERN coordinator, as an ERN and/or country can be selected as an entry point. The matrix tool shows (i) all relevant university and regional sites per country, (ii) if they are included in the specific ERN, (iii) inclusion in any other ERN, (iv) if they are included in the c4c national network and (v) if they are a university or regional hospital. A use-case screenshot is shown in Figure 8. With the example of Denmark, a coordinator and/or project manager of ERKNet can see that two-university hospital are included within the ERN. Three additional university hospitals are connected with the c4c national research network, of which two have experience working in another ERN. Additionally, eleven regional hospitals are connected with the c4c network with a paediatric clinical trial availability.

### 3.3 Tool and heatmap interviews

A total of 17 interviews were conducted including 27 unique representatives of an ERN. From the representatives, 59% (*n* = 16) were ERN project managers, 37% (*n* = 10) were ERN coordinators and 4% (*n* = 1) was a HCP representative. The interviews cover 74% of the paediatric-oriented ERNs, including ERN Paedcan (*n* = 22).

Results of the thematic analysis are included in Table 3. The heatmap and tools are praised for their effectiveness in identifying capable sites and fostering collaboration across networks, thereby benefiting both c4c and ERN sites. An example response from an ERN project manager includes, “*(the heatmap and matrix) are very well organized and it's obvious (to the user) how to get the information (on the ERNs and c4c overlap), so that can facilitate participation in clinical (for ERNs).”* Furthermore, they provide valuable operational insights that support the pharmaceutical industry and patient care improvements.

**Table 3:** Thematic analysis of the ERN representatives’ interviews (*n* = 27 representatives).

Thematic mapping	Commentary on heatmap and matrix tool	Suggestions for future optimalisation
Implications for research and collaboration	- The mapping and matrix have been commended for their **effectiveness** in pinpointing sites that are **equipped to conduct pediatric clinical trials**. - They serve as valuable tools for **fostering cooperation** between different networks. - The identification of overlaps at the site level is **mutually** beneficial: ○ It assists the c4c in identifying which sites are also involved with ERNs. ○ Conversely, being part of c4c can act as an additional mark of quality for an ERN site.	- N/A
Operational insights and suggestions for improvement	- The analysis offers **practical insights** into the **functioning** of ERNs (European Reference Networks) and the c4c (conect4children) network. - It underscores the usefulness of these analytical tools in aiding the **pharmaceutical** industry's operations and improving patient care.	- **Operational strategies** for enhancing the matrix include: ○ Regular updates to keep pace with changes in ERN membership. ○ Incorporation of detailed information about the specific areas of expertise for each site. ○ Extension of the mapping to cover the involvement of experts at each site. - Implementing a strategy for the **transfer of code** and source data to both networks would be advantageous for ongoing maintenance.
Structural limitations and network integration	- The analysis identifies **structural limitations** within the European Reference Networks (ERNs), specifically in the context of including **university** centers. - It is highlighted that, due to the current European structural setup, not all university centers are eligible or included in the ERNs.	- The findings suggest there is a need with the ERNs for: ○ A more **inclusive approach** to integrate centers within the ERNs. ○ Potential reevaluation of the **criteria for inclusion** and collaboration to allow more comprehensive coverage. - Emphasis is on ensuring the capability of the ERN network to potentially conduct trials for rare to ultra-rare diseases, which is crucial in pediatric care.
Data accessibility and utilization concerns	- N/A	- The analysis underscores the importance of exporting data into **user-friendly formats**, such as Excel, for better analysis of which sites are part of the ERN and to understand their capabilities. - Concerns have been expressed about the possibility that openly accessible matrices could reveal sensitive information to other stakeholders undermining incentive to use facilitation networks. - Open access of the matrix enforces concern that such exposure of data might be disadvantageous to hospitals with smaller patient populations
Clinical trial preparedness and experience	- The mapping and tool used in the analysis are **limited** to comprehensively record **specific details about clinical trial experience and the quality of each site.**	- This limitation highlights the need to **involve national networks** in the interpretation of data and in verifying the **current status** of the sites.
specialty networks and subspeciality mapping	- The matrix does **not currently contain** data on **subspecialty networks**, which are critical for the efficiency and effectiveness of pediatric clinical trials. - This is particularly relevant for areas such as rheumatology, where subspecialty network expertise is essential. - The inclusion of these subspecialty networks would provide a more detailed perspective of the clinical trial landscape for rare diseases.	- To enhance the mapping of rare disease clinical trials, it is recommended to include subspecialty networks such as PRINTO and ESCAPE.
Specific challenges in oncology and surgical research	- The existing tools are not suitable for mapping paediatric oncology trials and therefore should not be included in the current analysis.	- The surgical focus of certain ERNs calls for more detailed considerations in the mapping process, particularly concerning transplant capabilities.

ERN, European Reference Network; c4c, conect4children; PRINTO, Paediatric Rheumatology International Trials Organization; ESCAPE, European Paediatric Kidney Research; N/A, not available. Bold highlights keywords.

Alongside these positive aspects, the interviews also offer constructive optimization points for further enhancement of these tools and networks.

## 4 Discussion

**Figure 9:**
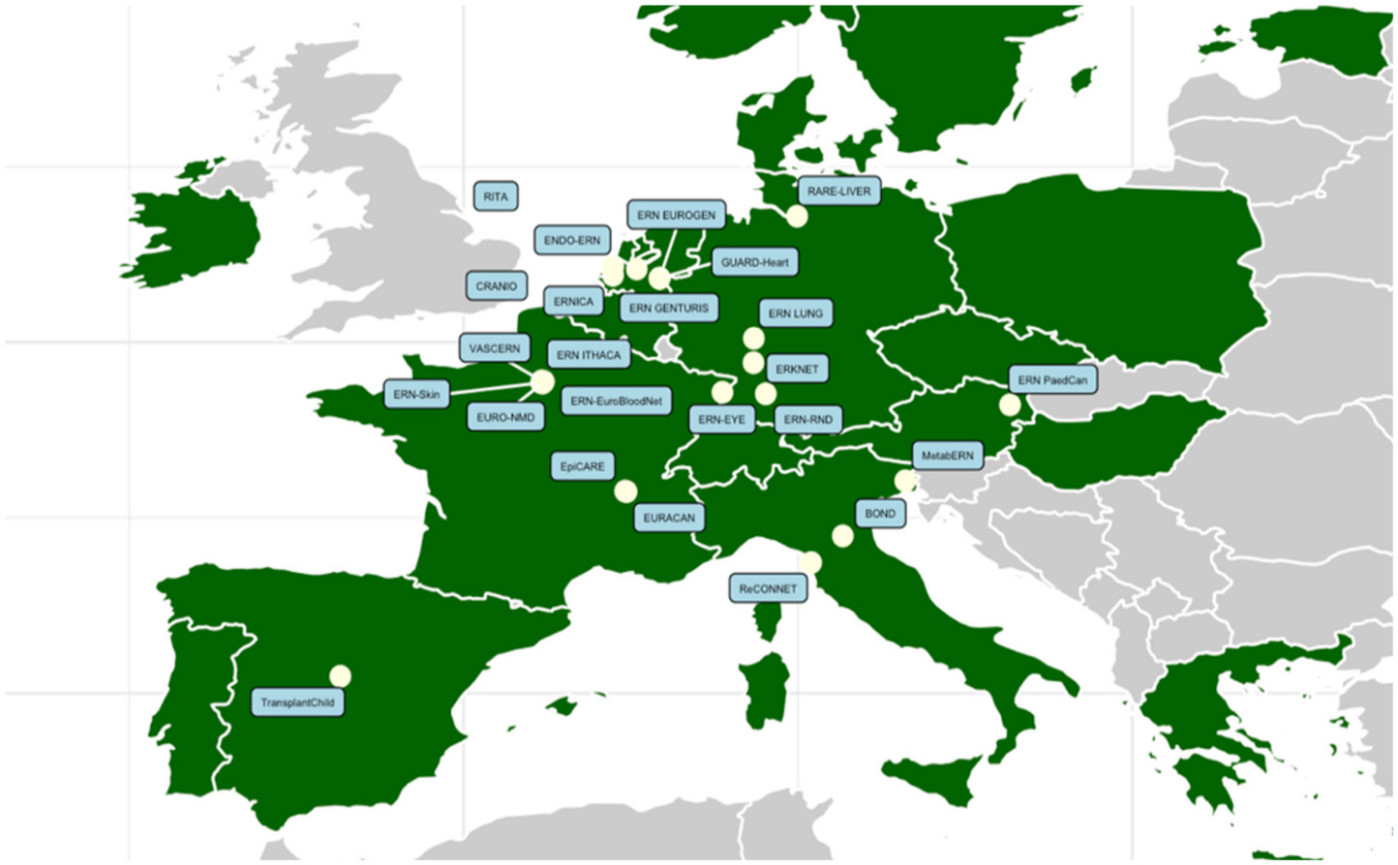
Location of the coordination offices per ERN in the overlapping countries within both c4c and ERNs. c4c, conect4children consortium; ERN, European Reference Network. Created within RStudio (v2023.12.0). For ERN acronyms please see Table 1.

Despite being designed for different purposes—ERNs focusing on subspecialty related clinical care and research in children and adults and c4c on interventional trials in children—the heatmaps reveal a significant overlap between the two networks, especially in Western European countries. The highest consistent overlap is observed in the Netherlands, Belgium, Sweden, Denmark, and the Czech Republic (see Figure 9). Interestingly, while Germany, France, and Italy are reported by Mordor Analytics (2023) to have the highest market value for paediatric clinical trials in Europe, the overlap in clinical trial preparedness for rare diseases is not as prominent in France and Italy. Meaning while these countries may lead in market size, their integration and readiness for rare disease trials within the ERNs and c4c networks need further development (32).

The comparison of the two heatmaps 5 and 6 reveals that nearly all locations within the ERNs that have a paediatric representative are also part of the paediatric network included within c4c, highlighting the potential for collaboration between these networks in supporting paediatric research. However, the overlap varies significantly across different ERNs. For instance, ERN ITHACA, Endo-ERN, and ERKNet consistently show a high overlap with the c4c paediatric network, indicating strong integration with paediatric sites and likely reflecting their focus on conditions affecting children. In contrast, ERN ReCONNET, ERN Skin, and ERN EuroBloodNet exhibit low overlap, which may be due to their focus on adult presentations of their respective thematic areas.

Additionally, while the integration is strong at a general level, only about one third of the c4c university sites are involved per ERN. This discrepancy suggests that not every university site offers treatment for every rare disease (RD) or are providing the care but are not connected to an ERN (due to workload, budget, restrictions in admission), highlighting the need for broader national network involvement to facilitate RD-focused paediatric trials and to optimize recruitment. Recruitment of all eligible patients across the country is essential to ensure timely enrollment. By understanding the complementary between c4c network and ERN-sites, stakeholders can better strategize to enhance paediatric trial networks, ensuring comprehensive coverage and support for rare disease treatments.

Moreover, one of the crucial factors in the success of a clinical trial is recruiting a sufficient number of participants to ensure a meaningful response to the trial's primary analysis (33, 34). The matrix can help ERN or c4c coordinators identify capable sites for interventional trials and foster network collaboration when approached by trial sponsors. Recent initiatives like IHI Moonshot and Together4RD underscore the importance of these efforts for enhancing trial efficiency and reach (35, 36).

In terms of interventional clinical trials, access to main university sites and regional sites that see people living with RD is important (33), especially considering countries with geographical difficulty to reach reference sites such as the Nordic (namely Sweden and Denmark), Eastern and Southern countries with a recurring lower availability of overlap and overall ERN sites. Median regional inclusion ratio is lower for ERNs than for the paediatric networks. Regional sites are important in interventional trials when considering different geographical distributions and burden of trials on children and their parents and for this reason could potentially be incorporated in a decentralised trial model (37–40). Decentralised trials are trials where trial activities are performed at a participants’ home and/or at local health care facilities, and have been the focus of many initiatives such as European Innovative Health Initiative (IHI) funded project Trials@Home (41, 42). The c4c national coordination centres can aid in the facilitation of a higher site inclusion per country according to the heatmap and interactive matrix tool, evaluated positively by the ERN-coordinators and project managers.

Future updates and maintenance of the matrix should focus on enhancing its dynamic capabilities to reflect the evolving landscape of ERNs and the c4c network. Adding granularity to the matrix by including detailed information about each site's areas of expertise and the involvement of experts will improve its applicability. Moreover, integrating data on subspecialty networks like PRINTO and ESCAPE can substantially improve the mapping's comprehensiveness in the rare disease landscape. Notably, some subdiscipline networks, such as PRINTO and the ESCAPE network, already have established collaborations with c4c, which can serve as a foundation for further integration (43).

To ensure clinical trial preparedness, national networks should be more involved in data interpretation and site status verification. Other limitations of this paper include the exclusion of at least one specific ERN due to unresponsiveness, the lack of explicit information about experts connected in c4c, and the availability of site-specific trial conduct metrics. Additionally, potential inaccuracies inherent in using self-reported data for creating heatmaps and coordination tools are noted.

## 5 Conclusion

This paediatric site mapping exercise serves as a developed tool to highlight the potential for complementarity, dialogue, and shared work between European Reference Networks and c4c networks. It promotes a collaborative framework, focusing on leveraging shared resources and expertise across regions. Future matrix updates should incorporate detailed site expertise.

## Data Availability

The raw data supporting the conclusions of this article will be made available by the authors, without undue reservation.
